# Adenosine accelerates the healing of diabetic ischemic ulcers by improving autophagy of endothelial progenitor cells grown on a biomaterial

**DOI:** 10.1038/srep11594

**Published:** 2015-06-25

**Authors:** Wen Chen, Yangxiao Wu, Li Li, Mingcan Yang, Lei Shen, Ge Liu, Ju Tan, Wen Zeng, Chuhong Zhu

**Affiliations:** 1 Department of Anatomy, National& Regional Engineering Laboratory of Tissue Engineering, Key Lab for Biomechanics and Tissue Engineering of Chongqing, Third Military Medical University, Chongqing 400038, China; 2 Department of Anatomy, Qiqihar Medical University, Qiqihar, China

## Abstract

Endothelial progenitor cells (EPCs) seeded on biomaterials can effectively promote diabetic ischemic wound healing. However, the function of transplanted EPCs is negatively affected by a high-glucose and ischemic microenvironment. Our experiments showed that EPC autophagy was inhibited and mitochondrial membrane potential (MMP) was increased in diabetic patients, while adenosine treatment decreased the energy requirements and increased the autophagy levels of EPCs. In animal experiments, we transplanted a biomaterial seeded with EPCs onto the surface of diabetic wounds and found that adenosine-stimulated EPCs effectively promoted wound healing. Increased microvascular genesis and survival of the transplanted cells were also observed in the adenosine-stimulated groups. Interestingly, our study showed that adenosine increased the autophagy of the transplanted EPCs seeded onto the biomaterial and maintained EPC survival at 48 and 96 hours. Moreover, we observed that adenosine induced EPC differentiation through increasing the level of autophagy. In conclusion, our study indicated that adenosine-stimulated EPCs seeded onto a biomaterial significantly improved wound healing in diabetic mice; mechanistically, adenosine might maintain EPC survival and differentiation by increasing high glucose-inhibited EPC autophagy and maintaining cellular energy metabolism.

Diabetes mellitus is a common chronic metabolic disease worldwide. The number of people with diabetes reached 366 million in 2011, and diabetes has become a substantial social and economic burden^1^. Diabetic foot disease is one of the most common complications of diabetes mellitus; the associated ulcers are usually accompanied by severe infections, ultimately causing death in a large number of diabetic patients^2^. Peripheral vascular impairment is considered to be a common cause of this disease, and it progresses even when the patient’s blood glucose level is under control^3^,^4^. High blood glucose levels and diabetic vasculopathy lead to reduced blood supply to the distal limbs, causing local skin ischemic ulcers to easily occur, further accelerating the progression of diabetic ulcers. Therefore, the current trend in treating diabetic ulcers is to restore the blood supply to the affected area and to maintain the normal supply of nutrition and oxygen to the surrounding cells.

EPCs have been defined as a type of stem cell that can colonize an ischemic site and differentiate into endothelial cells to promote angiogenesis^5^. However, the effect of stem cells in the absence of biomaterials is limited due to the great loss of cells. Researchers have transplanted EPCs to diabetic wounds after seeding the cells onto biomaterials; the results showed that this stem cell–biomaterial therapy considerably improved ischemic wound healing^6^,^7^,^8^. However, this effect was not significant when the transplantation was performed on diabetic ischemic wounds. One explanation for this might be that the poor microenvironment of diabetic wounds made it extremely difficult for EPCs to survive or differentiate, preventing EPCs from effectively functioning to promote angiogenesis^7^. It is well known that autophagy plays an important role in cellular homeostasis through the degradation and recycling of organelles such as mitochondria or the endoplasmic reticulum (ER) that are closely related to the pathogenesis of diabetes^9^. The influence of the diabetic ischemic microenvironment on the autophagy of transplanted EPCs and whether increasing autophagy helps to maintain EPC function and survival still require further investigation.

Adenosine is a common messenger that functions mainly through four G protein-coupled receptors, A1, A2a, A2b and A3^10^. Under conditions of energy starvation or environmental stress, the local adenosine concentration may correspondingly increase to regulate local cellular energy metabolism and to protect cells from damage. Therefore, adenosine is also referred to as a “retaliatory metabolite^11^”. Interestingly, autophagy also increases during cellular stress or starvation. Moreover, autophagy plays a crucial role in cell protection and in the control of energy balance, which is the same as the effect of adenosine. All of these findings suggest the possibility that adenosine exerts its cell protective effects through autophagy.

Consequently, stem cell–biomaterial therapy is the most promising treatment for diabetic wounds that has emerged in recent years. Adenosine can protect cells from damage, and many studies have shown that adenosine receptor activation can effectively promote diabetic wound healing^12^,^13^,^14^. Therefore, we investigated whether adenosine can promote diabetic ischemic wound healing by increasing autophagy in EPCs grown on biomaterials. To confirm our hypothesis, we generated a degradable biomaterial with high biocompatibility and then seeded this material with adenosine-stimulated EPCs and transplanted it onto diabetic ischemic wound surfaces to improve diabetic wound healing.

## Results

### Inhibition of EPC autophagy in diabetes

**Figure 1:**
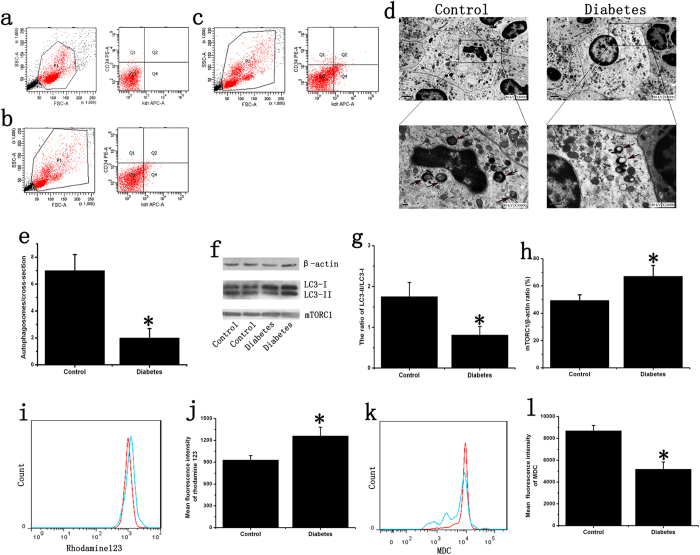
The inhibition of EPC autophagy in diabetes. (**a**) Mononuclear cells isolated from non-diabetic people, as a negative control. (**b**) Mononuclear cells isolated from non-diabetic people were labeled with PE-CD34 and APC-VEGFR2/KDR (Flk-1) antibodies. Double-positive cells were isolated by sorting on a FACSAria II SORP. (**c**) Mononuclear cell isolated from diabetic patients were labeled with PE-CD34 and APC-VEGFR2/KDR (Flk-1) antibodies. (**d**) TEM confirmed the presence of autophagosomes in the EPCs; arrows were used to show the autophagosomes. (**e**) Quantitative analysis of the number of autophagosomes in the control and diabetes groups (n = 10, *p < 0.05). (**f**) The inhibition of EPC LC3-I to LC3-II conversion and the increase of EPC mTORC1 in diabetes. We used cropped gels; the gels were run using the same experimental conditions. (**g,h**) The expression of LC3-I, LC3-II and mTORC1 in the control and diabetes groups. β-actin was used as a loading control (n = 10, *p < 0.05). (**i,j**) The mean fluorescence intensity of rhodamine 123 in the control and diabetes groups. The mitochondrial membrane potential of EPCs in the diabetic patients was higher than that in the healthy people (n=10, *p < 0.05). (**k,l**) The mean fluorescence intensity of MDC in the control and diabetes groups. The level of autophagy in the diabetic patients was much lower than that in the healthy people (For Fig. 1i and k, red = non-diabetic control subjects; blue= diabetic patients) (n = 10, *p < 0.05).

We directly sorted EPCs from peripheral blood, and transmission electron microscopy (TEM) results revealed a significant reduction of autophagosomes per cellular cross-sectional area in diabetic patients (Fig. 1a–e). Moreover, western blot results showed that the LC3 ratio (LC3-II/I) of EPCs was inhibited to approximately 53% in EPCs from patients with diabetes compared with those from non-diabetic controls. MTORC1 over expression could inhibit autophagy, and our results showed that mTORC1 expression was increased in diabetic patients (Fig. 1f–h). Moreover, we determined the mitochondrial membrane potential (MMP) of peripheral blood EPCs. The results demonstrated higher MMP in diabetic patients; MMP was increased by up to 30% compared with that in healthy subjects (Fig. 1i,j), while the fluorescence intensity of the autofluorescent marker monodansylcadaverine (MDC) was decreased by almost half (Fig. 1k,l). The results shown in Fig. 1 were obtained from a representative sample, and the quantification was based on the whole sample (n = 10). These results suggest that the imbalance between EPC autophagy and energy metabolism occurred in diabetic patients.

### Effects of adenosine on late EPC autophagy and maintenance of cellular energy metabolism

**Figure 2:**
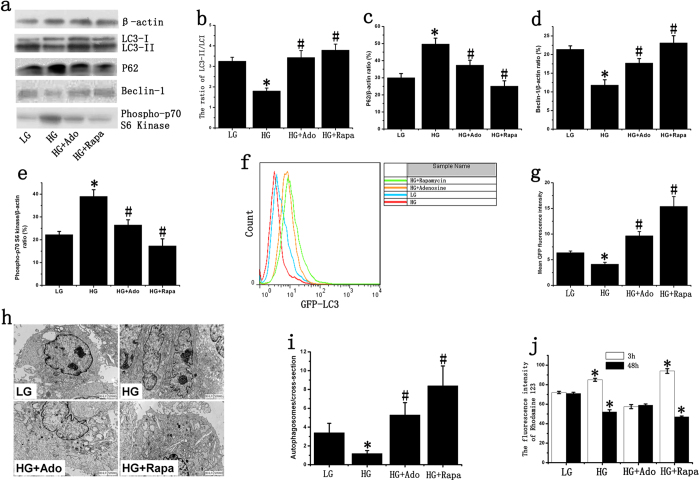
Effect of adenosine on late EPC autophagy. (**a**) LC3, P62, Phospho-p70 S6 Kinase and Beclin-1 were determined by western blotting. We used cropped gels; the gels were run using the same experimental conditions. (**b–e**) The expression of LC3-I, LC3-II, P62, Beclin-1 and Phospho-p70 S6 Kinase in late EPCs. β-actin was used as a loading control. (**f**,**g**) The mean LC3-GFP fluorescence intensity of late EPCs. The mean LC3-GFP fluorescence intensity was decreased after stimulation with HG, and adenosine could increase HG-inhibited autophagy. (**h**) TEM confirms the presence of autophagosomes in the late EPCs. (**i**) Quantitative analysis of the number of autophagosomes in late EPCs. (**j**) The mean fluorescence intensity of rhodamine 123 in late EPCs. The cells were stained with rhodamine 123 after 3 or 48 hours of stimulation, and the average fluorescence values were analyzed using flow cytometry. The groups were as follows: low glucose (LG); high glucose (HG); high glucose + adenosine (HG+Ado); high glucose + rapamycin (HG+Rapa). *p < 0.05 (n = 4) versus LG. #p < 0.05 (n =4) versus HG. Values are the mean ± SE.

Western blot results indicated that expression of the EPC autophagy markers LC3-II/I and Beclin-1 at physiological glucose concentrations decreased by 44.5% and 54.28%, respectively, after stimulation with high glucose (HG). After the addition of adenosine, LC3-II/I and Beclin-1 expression increased by approximately 107% and 84.9%, respectively. The levels of LC3-II/I and Beclin-1 were highest in the group treated with the mTOR inhibitor rapamycin and were increased by 1.97-fold and 1.57-fold, respectively, relative to the HG group (Fig. 2a,b,d). Stimulation with HG could increase P62 and Phospho-p70 S6 Kinase expression, and adenosine could inhibit HG-induced P62 and Phospho-p70 S6 Kinase expression (Fig. 2c,e). Subsequent GFP-LC3 flow cytometry in late EPCs confirmed our western blot results and showed that adenosine increased HG-inhibited EPC autophagy (Fig. 2f,g). The quantification of autophagosomes revealed a significant reduction after stimulation with HG. However, adenosine treatment enhanced the autophagic activity of EPCs, which was inhibited by HG (Fig. 2h,i).

We also examined the levels of energy metabolism *in vitro*. The results showed that, relative to the control group, EPC MMP increased by 18%, 3 hours after the addition of HG. Adenosine treatment decreased HG-induced MMP and was approximately 67.4% of the MMP in the HG group. The highest level of MMP was observed in the mTOR inhibitor group, in which MMP increased by 63.4%. After 48 hours, the level of MMP significantly decreased in the HG group, whereas in the mTOR inhibitor group, MMP stimulation continued, but some cell death was observed. Meanwhile, no significant change was observed in the adenosine group (Fig. 2j). Flow cytometry results showed that HG could induce EPC apoptosis and that adenosine treatment decreased HG-induced cell apoptosis (Fig. S2). However, no significant difference in overall EPC proliferation was observed between the groups (Fig. S3).

### Late EPC growth on the biomaterial

**Figure 3:**
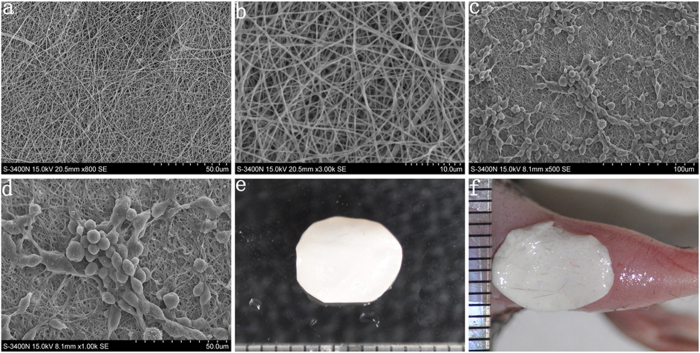
EPC growth on the biomaterial. (**a,b**) The structural characteristics of the biological material. Electrospinning was utilized to produce the biological tissue material, and SEM was used to observe the structural characteristics of the biological material. (**c,d**) The morphology of EPCs grown on the biomaterial. SEM results showed that EPCs grew well and formed EPC colonies with surrounding spindle-like cells on the material. (**e**) General image of the biomaterial. (**f**) The biomaterial seeded with EPCs was transplanted on the diabetic wound.

We cultured EPCs on our biomaterial and demonstrated via scanning electron microscopy (SEM) that the EPCs grew well and that EPC colonies with surrounding spindle-like cells appeared on this material, indicating that the biomaterial was suitable for EPC growth (Fig. 3c,d). Flow cytometry results showed that the biomaterial had no apparent effect on late EPC differentiation (Fig. S4)

### Functions of adenosine in late EPC survival

**Figure 4:**
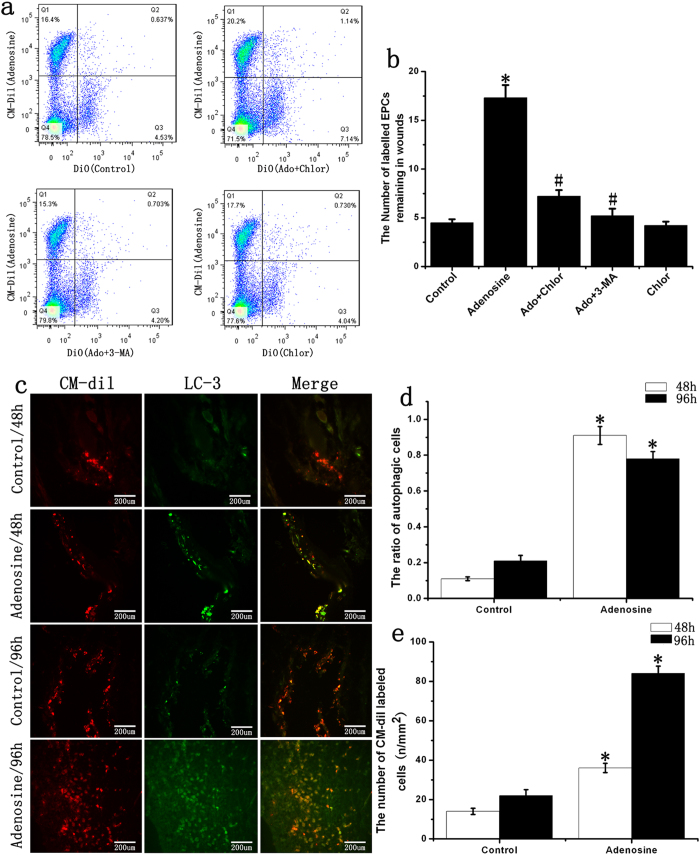
Function of adenosine on late EPC autophagy *in vivo*. (**a**,**b**) The number of labeled EPCs remaining in the wounds. The number of labeled EPCs was analyzed by flow cytometry 48 hours after transplantation. Adenosine could increase the number of EPCs remaining in the wounds, and chloroquine (chlor) or 3-MA could decrease the number of labeled EPCs remaining in the wounds. (**c**) Late EPCs were stained with CM-Dil (red) and were transplanted for 48 or 96 hours. The cells were stained with LC3 antibody (green). (**d**) The ratio of autophagic cells in diabetic ischemic wounds at both 48 and 96 hours. (**e**) The number of CM-Dil labeled EPCs remaining in diabetic ischemic wounds. *p < 0.05 (n = 6) versus control. #p < 0.05 (n = 6) versus adenosine. Values are the mean ± SE.

Forty-eight hours after transplantation, the number of labeled EPCs remaining in the wounds in the adenosine group was 3.62-fold higher than the number in the control group; additionally, the inhibition of autophagy could inhibit the adenosine-induced increase in the number of cells remaining in wounds (Fig. 4a,b). To investigate the role of adenosine in EPC autophagy, we used immunofluorescent techniques. The experimental results showed that adenosine significantly increased EPC autophagy; the autophagy rate was 8-fold higher than that of the control group 48 hours after transplantation and approximately 3-fold higher after 96 hours (Fig. 4c,d,e).

### Functions of adenosine on late EPC differentiation

**Figure 5:**
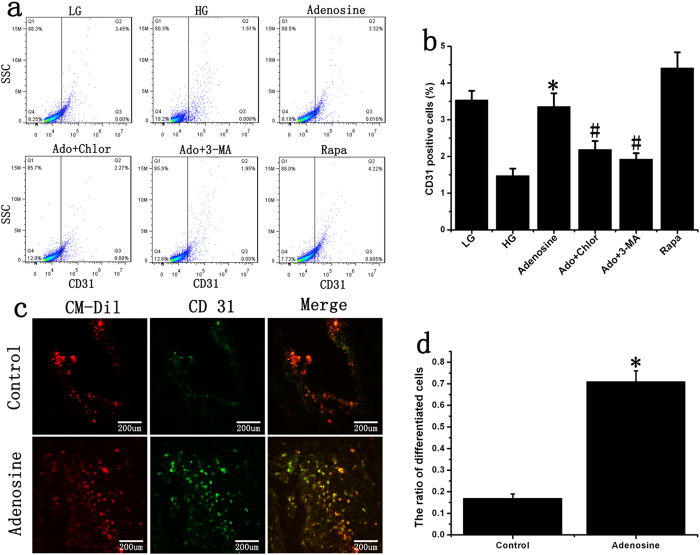
Effect of adenosine on late EPC differentiation. (**a,b**) The detection of CD31-positive cells. Adenosine promoted late EPC differentiation *in vitro*. The autophagy inhibitor reversed adenosine-induced EPC differentiation, while the mTOR inhibitor markedly enhanced the differentiation of EPCs into endothelial cells. *p < 0.05 (n = 6) versus HG. ^#^p < 0.05 (n = 6) versus adenosine. Values are the mean ± SE. (**c,d**) The ratio of differentiated EPCs in diabetic ischemic wounds. Late EPCs were stained with CM-Dil (red) and transplanted onto foot wounds in diabetic mice for 96 hours. The cells were stained with CD31 antibody (green). *p < 0.05 (n = 6) versus control. Values are the mean ± SE.

Flow cytometry revealed that adenosine promoted EPC differentiation. The autophagy inhibitors chloroquine and 3-Methyladenine (3-MA) reduced adenosine-induced EPC differentiation, whereas the mTOR inhibitor rapamycin markedly enhanced EPC differentiation into endothelial cells (Fig. 5a,b). The animal experiments also indicated that adenosine significantly promoted EPC differentiation on the surfaces of diabetic wounds (Fig. 5c,d).

### Stimulation of EPCs to promote diabetic wound healing by adenosine

**Figure 6:**
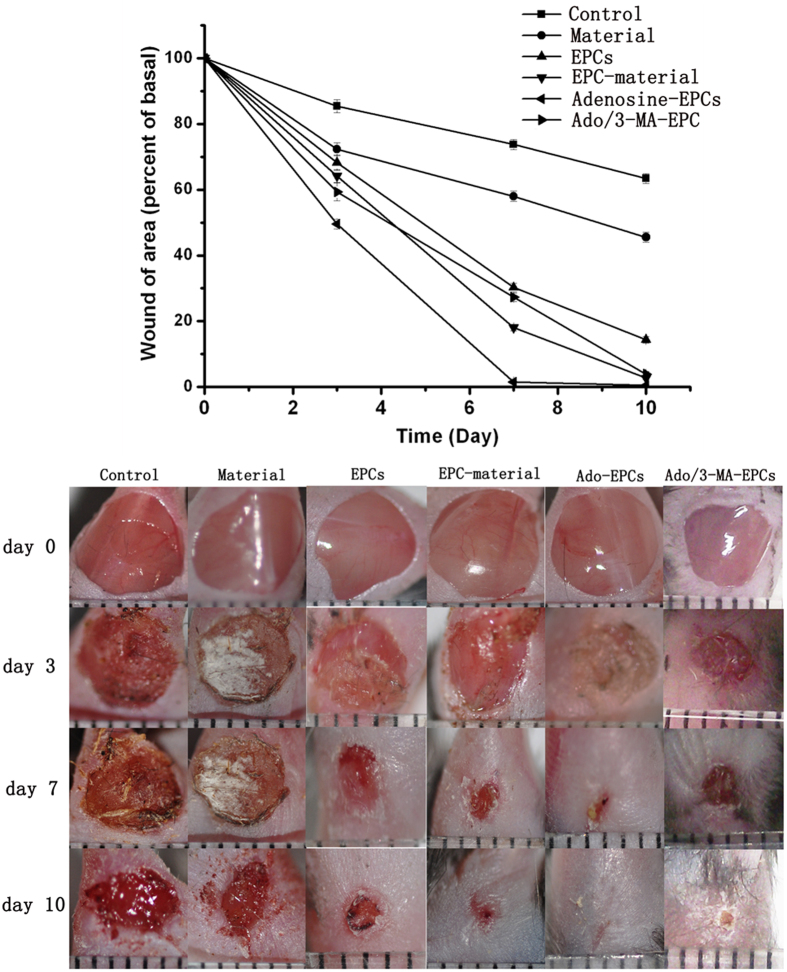
Stimulation of EPCs with adenosine to promote diabetic wound healing. (**a**) Adenosine-stimulated EPCs effectively promoted diabetic wound healing. Compared with the control group, the biological material alone and signal EPCs displayed faster wound healing rates. The adenosine-stimulated EPCs showed significantly improved wound healing compared with the normal EPC-Biomaterial group, and autophagy inhibition could inhibit the effect of adenosine on diabetic wound closure. (**b**) Photographic documentation of wounds at days 3, 7 and 10. The wound was very well healed on day 7 and was completely closed on day 10 in the adenosine-stimulated EPCs group. *p < 0.05 (n = 10) versus the control group. Values are the mean ±SE; #p < 0.05 (n = 10) versus the EPC-Biomaterial group.

From the day of surgery, we observed wounds in the diabetic mice on days 3, 7 and 10 and measured the areas of the wounds using IPP software. The results indicated that compared with the control group, the biological material alone displayed a faster wound-healing rate. Our material is a degradable biomaterial with high biocompatibility^15^,^16^, and the wound healing in biomaterial group was not fast enough to observe the process of material degradation. EPC-Biomaterial therapy displayed significantly accelerated wound healing compared with the signal EPCs group. The adenosine-stimulated EPCs significantly improved wound healing compared with the normal EPC-Biomaterial group, and there was a significant difference in the treatment effects. The wounds had healed very well by day 7 and were completely sealed by day 10 in the adenosine-stimulated EPC-Biomaterial group. The inhibition of autophagy could significantly inhibit the effect of adenosine on the closure of diabetic wounds (Fig. 6).

**Figure 7:**
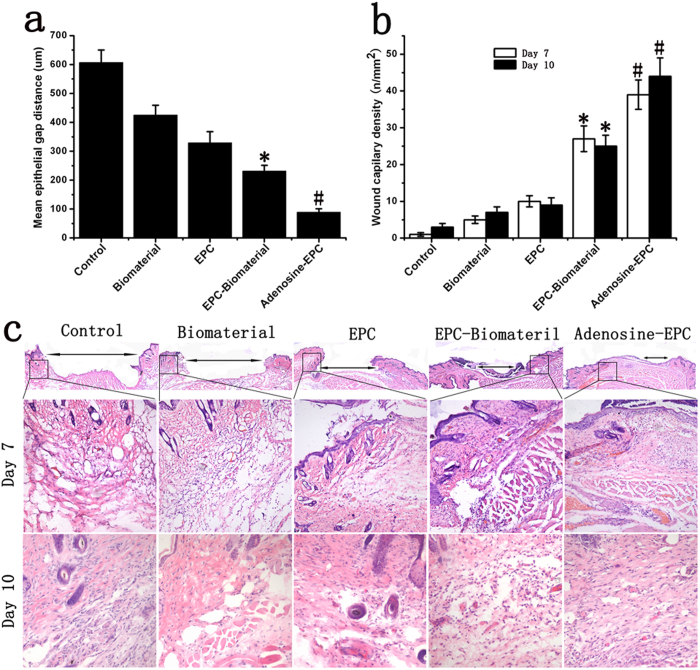
Effects of adenosine-stimulated EPCs on angiogenesis in diabetic wound. (**a**) Mean epithelial gap distance on day 7. (**b**) The wounds capillary density on day 7 and day 10. The analysis of wounds capillary density was based on H&E staining. The biological material and signal EPC group promoted angiogenesis compared with the control group. The material seeded with adenosine-stimulated EPCs increased the vascular density compared with the normal EPC group. (**c**) We performed H&E staining to observe the epithelial gap and vascular density in each group. Wounds treated with adenosine-stimulated EPCs seeded on the biomaterial had significantly reduced epithelial gaps compared with wounds treated with EPCs seeded onto the biomaterial on day 7. The highest vascular density was found in the adenosine-stimulated EPC group. *p < 0.05 (n = 10) versus EPC; #p < 0.05 (n = 10) versus EPC-Biomaterial. Values are the mean ± SE.

To quantitatively assess wound healing, we also measured the epithelial gap using HE staining at 7 days after transplantation. Wounds treated with the biomaterial did not display significantly accelerated re-epithelialization compared with control wounds, and wounds treated with signal EPCs showed epithelial gap reduction, to a certain degree. Wounds treated with EPCs seeded on the biomaterial had a significantly reduced epithelial gap compared with both signal EPC- and biomaterial-treated wounds. However, the maximum accelerated re-epithelialization rate was observed in wounds treated in the adenosine-stimulated EPCs-Biomaterial group, and the vascular density of these wounds was higher than that of wounds treated with EPCs seeded onto the biomaterial but not treated with adenosine (Fig. 7).

### Effects of adenosine-stimulated EPCs on angiogenesis in diabetic wounds

HE staining results showed that our biomaterial promoted angiogenesis and the vascular density of the wounds was 3.5-fold higher compared with the control group on day 10. Wounds treated with the biomaterial seeded with EPCs also had increased vascular density, which was 3.85-fold higher than that of wounds treated with the biomaterial alone. The highest vascular density occurred in the adenosine-stimulated EPC- Biomaterial group; in this group, the vascular density was increased by more than 76% compared with the EPC-Biomaterial group on day 10 (Fig. 7b,c). CD31 immunofluorescence detection also revealed that adenosine-stimulated EPCs could effectively promote angiogenesis in diabetic wounds (Fig. S5).

### Effects of adenosine on the survival of transplanted EPCs in diabetic wounds

**Figure 8:**
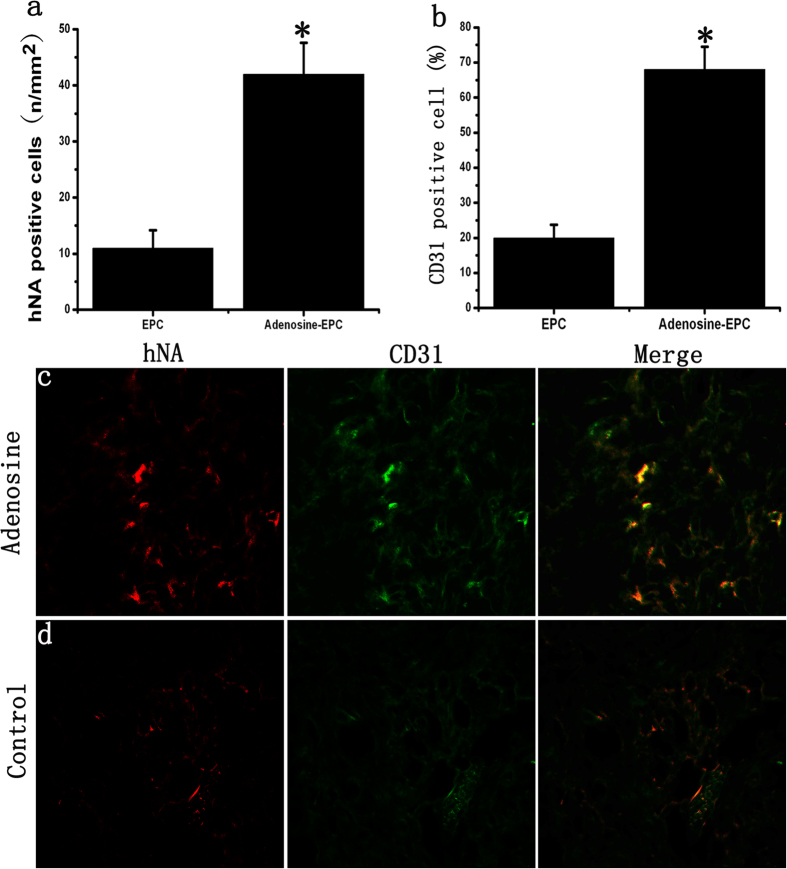
Effects of adenosine on the survival of transplanted EPCs in diabetic wounds. (**a**) The number of EPCs remaining in the diabetic wound of the adenosine-stimulated EPC group was 4.35-fold higher than that of the unstimulated EPC group. (**b**) The ratio of CD31-positive cells relative to the number of EPCs remaining in the diabetic wound. In the adenosine-stimulated EPC group, the number of remaining stem cells participating in angiogenesis was greater than that in the unstimulated EPC group. (**c**–**d**) Vascular endothelial cells were stained with a CD31 antibody (green), and human-derived cells were stained with an hNA antibody (red). *p < 0.05 (n = 10) versus control. Values are the mean ± SE.

On day 10 of wound healing, the number of EPCs remaining in the diabetic wounds of the adenosine-stimulated EPC group was 4.35-fold higher than that of the unstimulated EPC group; this difference was statistically significant (Fig. 8a,c,d). In the adenosine-stimulated EPC group, approximately 68.2% of the remaining stem cells participated in angiogenesis, whereas the participation rate was only 19.7% in the unstimulated EPC group (Fig. 8b,c,d). A TUNEL assay showed that the number of cells undergoing apoptosis in the adenosine-EPC group was significantly lower than that in the control-EPC group (Fig. S5).

## Discussion

A diabetic ulcer is a type of ischemic limb wound that is difficult to cure. Stem cell therapy and biomaterial technology have gradually become mainstream trends in the research and treatment of diabetic ulcers^17^,^18^. Due to the unique capacity of EPCs to promote angiogenesis, the use of EPCs to treat ischemic wounds has become a prominent area of research in recent years^7^,^8^. Our results showed that such cell therapy can promote the healing of diabetic wounds. However, the effect of stem cells without a biomaterial has been shown to be limited and temporary. This is mainly because EPCs are small cells; when they are transplanted to the surface of diabetic wounds, a substantial proportion of the cells quickly leave the wound^19^. Stem cell–biomaterial applications can increase cell retention and enhance EPC functions in diabetic wounds. Moreover, the physical microenvironments of biomaterials have been shown to play an important role in stem cell growth, survival and autophagy. Our prepared biomaterial was suitable for stem cell growth and survival, and this stem cell–biomaterial therapy significantly promoted wound healing in diabetic mice. This improvement may occur because the biomaterial can protect the transplanted cells at an early stage.

Autophagy is an important physiological phenomenon in multicellular organisms. During energy starvation or environmental stress, autophagy plays an important role in maintaining cellular homeostasis^20^. Our study found that the MMP of EPCs in patients with diabetes mellitus was higher than that in healthy subjects, but the level of autophagy was much lower. The increase in MMP indicates that the cells needed to obtain more raw materials for the ATP-synthesizing machinery and that they degraded more metabolic waste. However, in diabetic patients, the level of autophagy, which could degrade metabolic waste and transform it into materials for the ATP-synthesizing machinery, was reduced. If the reduction of autophagy lasted for a specified length of time, an increase of oxygen-derived free radicals and mitochondrial dysfunction would occur and could even result in cell death. As a result, the imbalance between autophagy and energy metabolism might be an important cause of cell injury and death in diabetes mellitus.

Similarly to autophagy, adenosine production is augmented in response to stress, and adenosine can ensure a normal supply of cellular ATP to meet the most basic energy requirements for cell survival^10^,^21^. Interestingly, we found that high extracellular adenosine decreased the level of cellular energy metabolism and increased EPC autophagy. The nature of autophagy is to remove metabolites and to release free substances, including free amino acids and fatty acids. These free substances are ultimately involved in the citric acid cycle to generate cellular ATP. Thus, after adenosine treatment, the decreased need for raw materials for ATP synthesis and the increased level of autophagy maintain EPC survival in diabetic wounds. Additionally, many studies have reported that autophagy plays an essential role in mammalian development and differentiation through the resulting production of necessary amino acids and protein catabolism^22^. Our results also confirmed that adenosine promoted EPC differentiation and that the inhibition of autophagy reversed adenosine-induced EPC differentiation. The differentiated cells directly participated in the process of neovascularization and finally promoted diabetic wounds healing.

To further validate our findings, we seeded our biomaterial with adenosine-stimulated EPCs, which were later transplanted onto the wounds of diabetic mice. On days 3, 7 and 10, this treatment promoted better wound healing compared with the transplantation of regular EPCs. Our staining results demonstrated that the adenosine-stimulated EPC group had higher levels of skin angiogenesis than the control group and that the number of EPCs remaining on the diabetic wound surfaces was also significantly higher. Therefore, we believe that adenosine increases EPC autophagy to prevent damage caused by the harsh external microenvironment of a diabetic wound and maintains the differentiation of EPCs, which are the two major mechanisms by which adenosine improves diabetic wound healing. The results of our study have great significance for the application of stem cell and biomaterial approaches to treating diabetic wounds and provide a better strategy for clinically treating diabetic foot disease.

In conclusion, our study indicated that adenosine-stimulated EPCs could significantly improve the healing of diabetic wounds; mechanistically, adenosine might maintain the survival and differentiation of EPCs grown on biomaterials by increasing high glucose-inhibited EPC autophagy and by maintaining cellular energy metabolism.

## Materials and Methods

### Ethics statement

This study was approved by the Ethics Committee of the Third Military Medical University and was performed according to the principles of the Declaration of Helsinki. The experiments involving human subjects were approved by Clinical Trail Ethics Committee of the Southwest Hospital of Third Military Medical University. All participants provided written informed consent, and the ethics committee approved the consent procedure.

### Cell labeling and sorting

This study included 10 patients with type 2 diabetes mellitus and 10 non-diabetic control subjects. Diabetes mellitus was diagnosed as fasting blood glucose ≥7.0 mmol/L and 2-hour post-load glucose ≥11.1 mmol/L. None of the patients had received prior treatment for diabetes or had cardiovascular complications. The diabetic patients were diagnosed at Southwest Hospital, affiliated with the Third Military Medical University (Chongqing, China), and their ages ranged from 40 to 55, regardless of sex. Non-diabetic controls, with no personal or family history of diabetes mellitus or other genetic diseases, were frequency matched to the diabetic patients for age and residential area (urban or countryside). The cloudy mononuclear cell layer was isolated from human peripheral blood and then labeled with PE-CD34 and APC-VEGFR2/KDR (Flk-1) antibodies. Double-positive cells were isolated by sorting on a FACSAria II SORP (BD Biosciences). In addition, we purified CD133-positive progenitor cells by positive selection with anti-CD133 microbeads using a magnetic cell sorter device (Miltenyi Biotec). Then, CD133-positive progenitor cells were divided into 2 groups: one group was labeled with the PE-CD34 antibody (Invitrogen) and the mitochondrial membrane potential (MMP) fluorescent dye Rhodamine 123 (150 ng/ml, Sigma); the other group was labeled with PE-CD34 antibody and the autofluorescent marker monodansylcadaverine (MDC, 0.05 mM, Sigma). The mean fluorescence value of cells was then measured using a flow cytometer (Becton Dickinson).

### Transmission electron microscopy (TEM)

The cells were fixed in 2% v/v glutaraldehyde for 24 hours. Samples were washed three times with PBS and cut according to standard procedures. Spherical structures with double-layer membranes in the EPCs were considered autophagosomes^23^. Imaging was performed on a TECNAI 10 TEM (Phillips). A total of 15–20 cellular cross-sections were counted for each sample.

### Western blotting

We extracted protein from EPCs with the RIPA Lysis Kit (Beyotime). The total protein concentrations were measured using the BCA Protein Assay Kit (Beyotime). All of the samples, each containing 30 g protein, were boiled with loading buffer for 5 min and then loaded onto a 12% SDS-PAGE gel for 1.5 h and transferred onto PVDF membranes. The membranes were blocked with 5% non-fat milk in TBST for 1 h and then incubated with primary antibodies against four autophagy markers, anti-P62 (Sigma), anti-LC3 (Sigma), phospho-p70 S6 Kinase, Beclin-1 and anti-actin, overnight. Next, we removed the excess antibodies by washing with TBST and incubated the membranes with horseradish peroxidase-coupled secondary antibodies for 1 h. The immunoblots were visualized using ECL chemiluminescence (Beyotime) and quantified with Quantity One software (Bio-Rad, USA).

### Cell culture and reagents

Peripheral blood was taken from healthy volunteer blood donors, and the cloudy MNC layer was isolated using density gradient centrifugation in human lymphocyte separation medium. Briefly, isolated mononuclear cells were cultured in a simple medium comprising M199 medium (HyClone), 10% fetal bovine serum (FBS, Gibco), 10 ng/mL vascular endothelial growth factor (VEGF; R&D Systems), 3 ng/mL basic fibroblast growth factor (bFGF; Roche Applied Science), and heparin (90 mg/mL; Sigma) in the presence of penicillin (100 Units/mL) and streptomycin (100 mg/mL) (all purchased from Sangon Co.). After 24 hours, the unattached cells were removed by washing with PBS, and the adhered cells were cultured. Two types of EPCs were isolated and cultured from adult peripheral blood, designated as early and late EPCs^24^. To obtain late EPCs, we continuously cultured the cells for longer than two weeks. The identity of the cultured EPCs was similar to that in our previous study (Supporting Information, Fig. 1)^25^. In the adenosine treatment group, we added 100 mmol/L of adenosine (Sigma). Prior to the addition of adenosine, 5 μmol/L of erythro-9-(2-Hydroxy-3-nonyl) adenine hydrochloride (EHNA, Sigma) was added to prevent adenosine degradation. After 14 days of culture, the EPCs were divided into four groups: low glucose (5.6 mM glucose), high glucose (30 mM glucose), high glucose with adenosine, and high glucose with adenosine and the mTOR inhibitor rapamycin (100 nmol/L, Sigma). Rapamycin, which is believed to activate autophagy, was used as the positive control in our experiments.

### Detection of EPC mitochondrial membrane potential (MMP)

We used Rhodamine 123 to detect the MMP. After 3 or 48 hours of stimulation, the EPCs were trypsinized with trypsin (HyClone), centrifuged, resuspended and stained with Rhodamine 123 for 20 minutes. After washing 3 times with PBS, the EPCs were analyzed using flow cytometry to detect the average value of fluorescence in each group.

### GFP-LC3 fluorescence intensity analysis

GFP-LC3 was used to assess autophagic flux. The late EPCs were transfected with GFP-LC3 by using X-treme GENE HP DNA Transfection Reagent (Roche) and then stimulated with adenosine. After 12 hours of stimulation, chloroquine was added to the medium. The cells were cultured for another 6 hours and then trypsinized. After washing 3 times with PBS, the EPCs were analyzed using flow cytometry to detect the mean LC3-GFP fluorescence intensity.

### Biomaterial preparation and EPC growth on the biomaterial

Polylactic acid (80%, Sigma), silk (10%) and type I collagen (10%, Sigma) were thoroughly mixed. Silk was prepared as the method reported by Lee *et al*^26^. In short, we degummed raw silk fibers twice with 0.5% NaHCO_3_ at 100 degrees celsius for 30 minutes and then washed 3 times. Degummed silk was then dissolved in CaCl_2_/CH_3_CH_2_OH/H_2_O for 6 hours and dialyzed with a cellulose tubular membrane (Sigma). After 3 days, the silk fibers were lyophilized to obtain degummed silk sponges. Electrospinning was utilized to produce the biological tissue material^15^,^16^. SEM was used to observe the structural characteristics of the biomaterial. After 14 days of culture, 2 × 10^4^ EPCs were seeded onto the biomaterial and cultured for 48 hours. SEM was also used to observe the state of cell growth.

### Animal experiments

All of the animals were handled according to the Guide for the Care and Use of Laboratory Animals of the Third Military Medical University (Chongqing, China) and were approved by the Ethics Committee of the Third Military Medical University. Eight-week-old male C57BL/6J mice (Experimental Animal Center of the Third Military Medical University) were intraperitoneally injected with 40 mg/kg streptozotocin (STZ; Sigma) for five consecutive days. Mice with a fasting blood glucose level >16.7 mmol/L were considered to be diabetic. The establishment of the diabetic foot model was accomplished as previously described^15^,^16^. The proximal femoral artery was ligated to induce ischemia in the lower limbs, and full-thickness wounds with diameters of 5 mm were generated in the dorsal skin of the thigh^7^. The wounds were covered with the biomaterial seeded with 2 × 10^4^ adenosine-activated EPCs or non-activated EPCs and later with a Tegaderm TM transparent dressing (Minnesota Mining and Manufacturing). After the surgery, the mice were supplied with adequate food and drinking water and were individually housed in an environment with constant temperature and humidity.

### Assays for monitoring late EPC autophagy and differentiation

Immunofluorescence staining was used to assess EPC autophagy and differentiation *in vivo*. The EPCs were divided into a control group and an adenosine group after 14 days of culture. A total of 2 × 10^4^ EPCs were stained with CM-Dil (Invitrogen) and seeded onto the biomaterial. The biomaterial was transplanted onto the foot wound of diabetic mice after being cultured for 48 hours. Then, 48 or 96 hours after transplantation, the skin tissue in the wound area was dissected, fixed in 4% paraformaldehyde and sectioned into 9-μm-thick slices by cryosectioning. We used LC-3 and CD31 (Abcam) antibodies for immunofluorescence labeling. A total of 5 cross-sections were counted for each sample and the number of CM-Dil label cells and positive staining cells were quantified per mm^2^. After being cultured for 14 days, the EPCs were divided into six groups: low glucose, high glucose, high glucose with adenosine, high glucose with adenosine and 20 μM of the autophagy inhibitor chloroquine or 10 mM 3-MA (Sigma), and high glucose with rapamycin. After 24 hours of stimulation, the cells were washed with PBS 3 times, and the culture medium was replaced with EPC medium containing 1% serum. After culturing for another 48 hours, the EPCs were trypsinized and stained with CD31-PE (Abcam), followed by 3 PBS washes. The EPCs were analyzed using flow cytometry to detect the average value of fluorescence in each group.

### Detection of EPCs in wounds

In our experiments, two methods were used to evaluate late EPC survival in diabetic wound. The first method was to directly observe the CM-Dil staining cells in the frozen section. The second method was performed as previously described^7^. Briefly, late EPCs were stained with CM-Dil (Invitrogen) in the adenosine group and with DiO (Sigma) in the control group, the adenosine and 3-MA, adenosine and chloroquine, and chloroquine alone groups. After being washed with PBS, the cells in the adenosine group were mixed with cells in the control or chloroquine group before transplantation. Two days after transplantation, the wounds were excised, cut into small pieces, digested in DMEM containing 0.2% collagenase II (Sigma) for 30 min at room temperature, washed in PBS 3 times and digested in PBS/2 mM EDTA/0.25% trypsin for another 10 min. The reaction was stopped by PBS/10% FBS, and the cells were then passed through a 76-μm filter. After spinning at 300 g for 5 min, the cells were analyzed by flow cytometry.

The skin tissue in the wound area was dissected at 7 and 10 days after transplantation, fixed in 4% paraformaldehyde and sectioned into 9-μm-thick slices by cryosectioning. Two antibodies were for immunofluorescence labeling: a CD31 antibody to mark vascular endothelial cells and an anti-human nuclear spliceosome antibody (hNA, Millipore) to mark human-derived cells. We also performed hematoxylin-eosin (HE) staining. We used an Olympus BX50 microscope to analyze the staining. We randomly chose five fields of view (400X), and VERSOI4.6 software was used to capture the images. Quantitative histological analysis of the capillary density of the wounds was also performed.

### Statistics

Each experiment was repeated at least four times. Analysis of variance and t-tests were performed using SPSS 18.0 (Version 18.0, SPSS Inc. Chicago IL, USA). All of the experimental data are expressed as the means ± SE; All p values were two-tailed, and a p value < 0.05 was considered statistically significant.

## Additional Information

**How to cite this article**: Chen, W. *et al.* Adenosine accelerates the healing of diabetic ischemic ulcers by improving autophagy of endothelial progenitor cells grown on a biomaterial. *Sci. Rep.* **5**, 11594; doi: 10.1038/srep11594 (2015).

## Supplementary Material

Supplementary Information
