# Supplementary material for: Adenosine accelerates the healing of diabetic ischemic ulcers by improving autophagy of endothelial progenitor cells grown on a biomaterial
Source: Sci Rep. 2015 Jun 25;5:11594. doi: 10.1038/srep11594 (PMC4479873; doi:10.1038/srep11594)

Adenosine accelerates the healing of diabetic ischemic ulcers by improving autophagy of endothelial progenitor cells grown on a biomaterial

Wen Chen, Yangxiao Wu, Li Li, Mingcan Yang, Lei Shen, Ge Liu, Ju Tan, Wen Zeng, Chuhong Zhu

Supplementary Material

1. Fluorescence-Activated Cell Sorter (FACS) Analysis

EPCs were analyzed by FACS. After 7 day culture, we used antibodies against human CD34 and VEGFR-2(KDR). Briefly, cells were resuspended in PBS with APC- VEGFR-2 or PE-CD34 antibody and incubated 30 minutes. Cells were washed three times and resuspended for FACS.

2. Apoptosis detection

After the cells were stimulated for 48 h, Annexin V protein and corresponding nucleic acid dyes were added, followed by 15 min of reaction in the dark at room temperature. Finally, after the addition of binding buffer, apoptotic cells were counted using a flow cytometer.

3. MTT assay

MTT was dissolved in PBS at 5 mg/ml and filter sterilized through a 0.2 m M filter. 4h before the end of the incubation, 20 ml of the MTT solution was added to each well, and the plates were incubated at for 4 h. The medium was removed and

150ml of DMSO was added to each well. After 15 min of oscillation, the plate was placed in an incubator for 5 min to eliminate air bubbles. On a plate reader, the absorbance at a wavelength of 490 nm was measured for each sample.

4. Identification of EPC growth on the biomaterial.

After 14 days of culture, EPCs were seeded onto the biomaterial and cultured for 48 hours. PE-CD34 and APC-VEGFR-2 antibodies were used to detect the characterization of late EPCs.

5. Detection of cell apoptosis

The specimens were washed with PBS for 30 min. The washed specimens were blocked with 3% H_2O_2 for 10 min and clarified with 0.1% Triton X-100 (Sigma) for 2 min. After washing with PBS three times, the TUNEL reaction mixture (Roche) was added to the sections, followed by incubation at 37°C for 60 min. Then, the specimens were stained with 4',6-diamidino-2-phenylindole (DAPI) (Sigma), washed with PBS three times, and mounted. The expression of the fluorescence was observed under a fluorescence microscope.

Supplementary Results

Figure S1. Identification of EPC phenotype. (a) After 3 days, attached cells appeared to be clusters. The cells were grown to confluence showing a cobblestone-like monolayer after 14 day culture. (b) The cells showed expressions of both CD34 and

VEGFR-2(KDR) antigens as analyzed by FACS after 7 day culture.

Figure 1

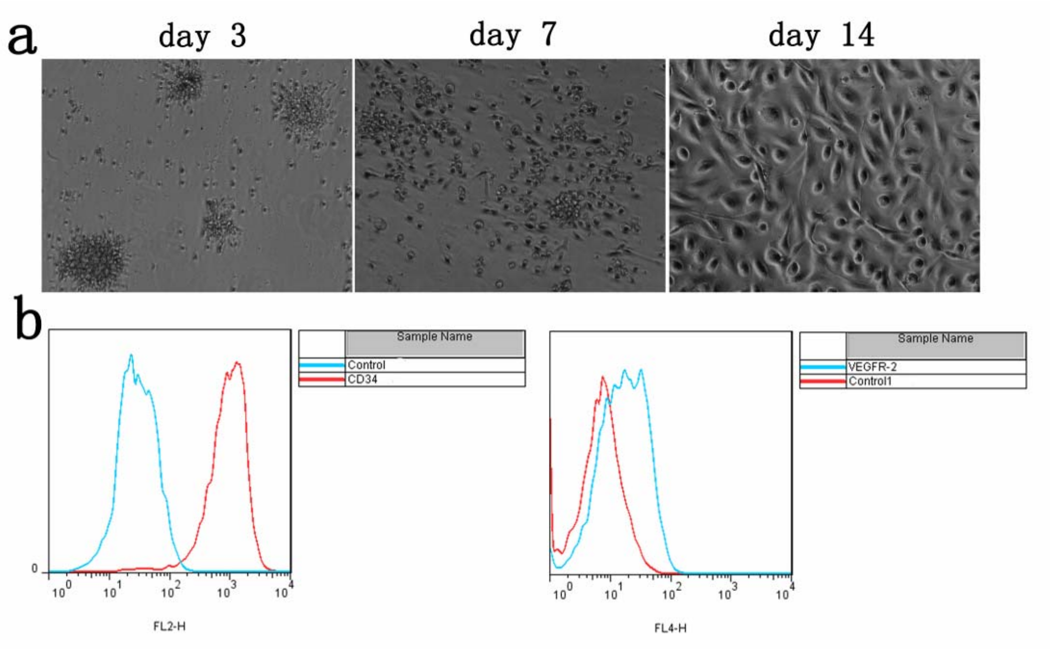

Figure S2. Adenosine inhibited HG-inducing EPC apoptosis.

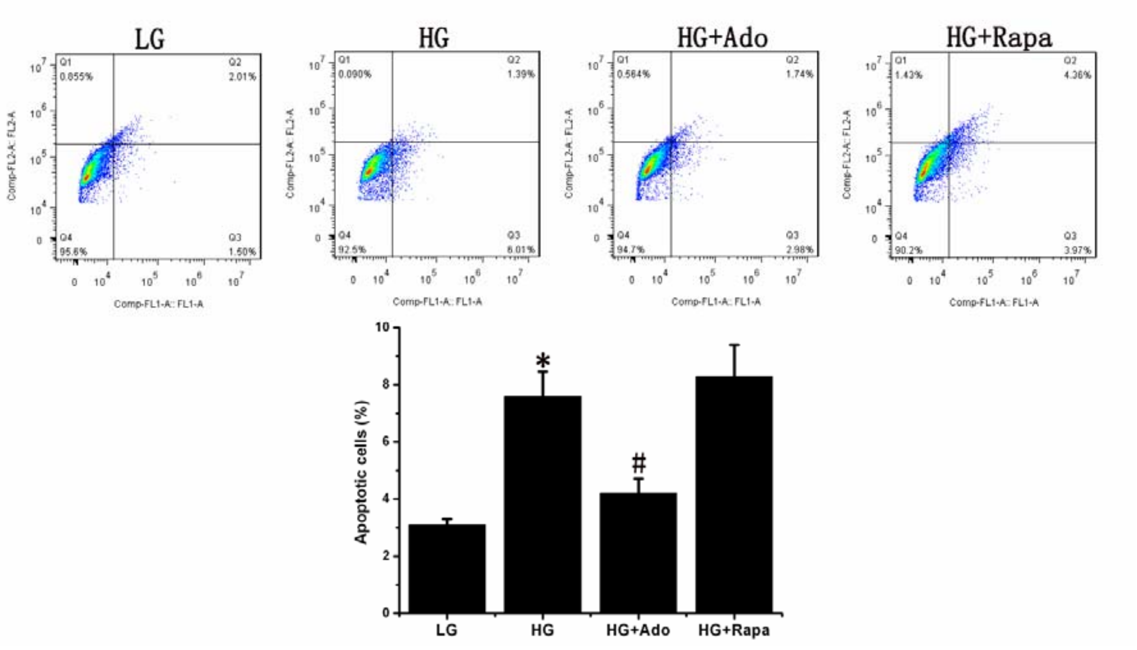

Figure S3. Adenosine had no significant effect on EPC proliferation.

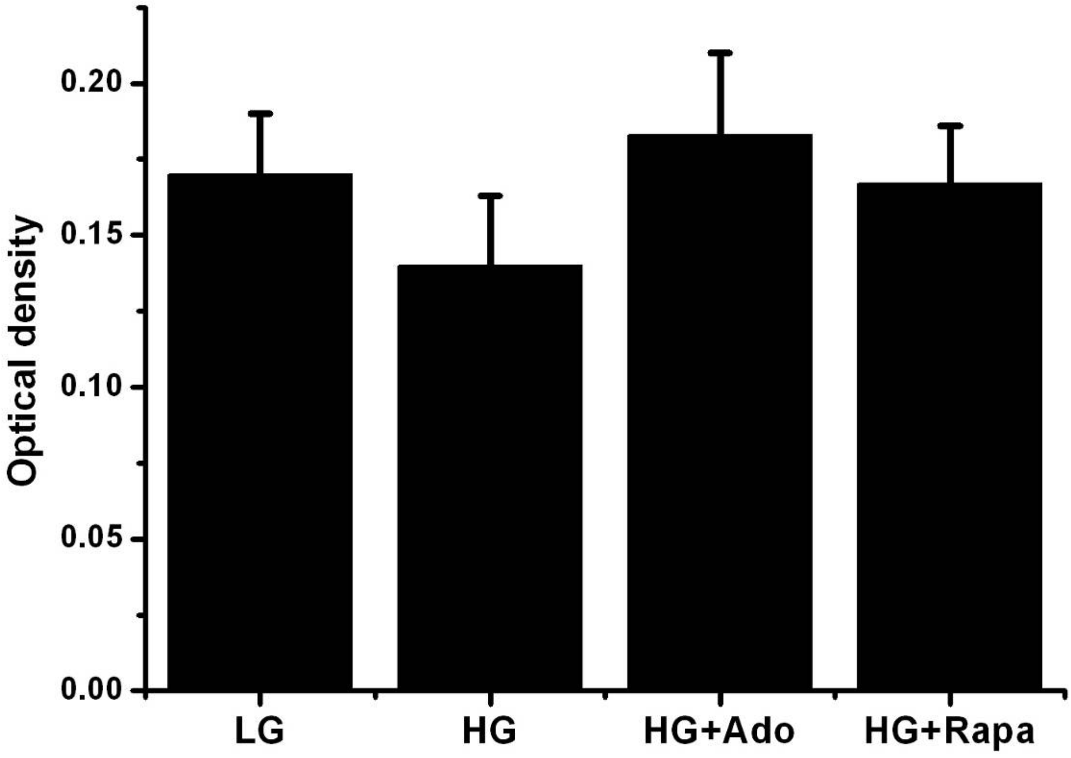

Figure S4. The cells showed expressions of both CD34 and VEGFR-2(KDR) antigens as analyzed by FACS after seeded on the biomaterials. The late EPCs untreated with PE-CD34 or APC-VEGFR-2 antibodies were used as the negative control.

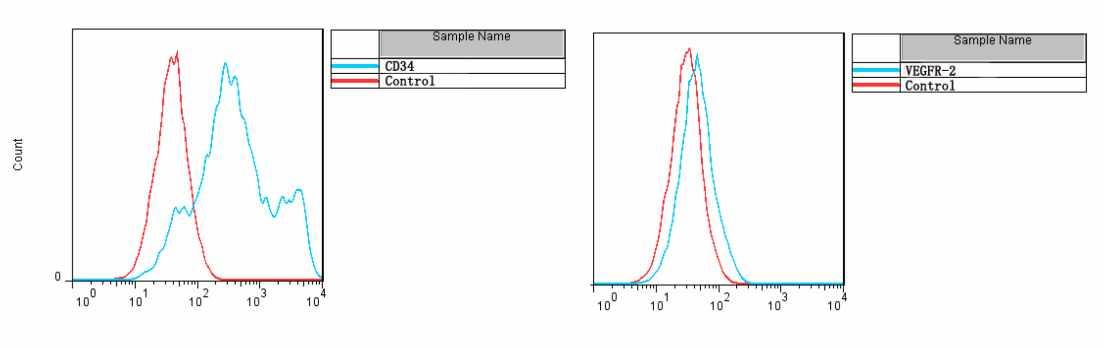

Figure S5. Effects of adenosine-stimulated EPCs on angiogenesis in diabetic wound.

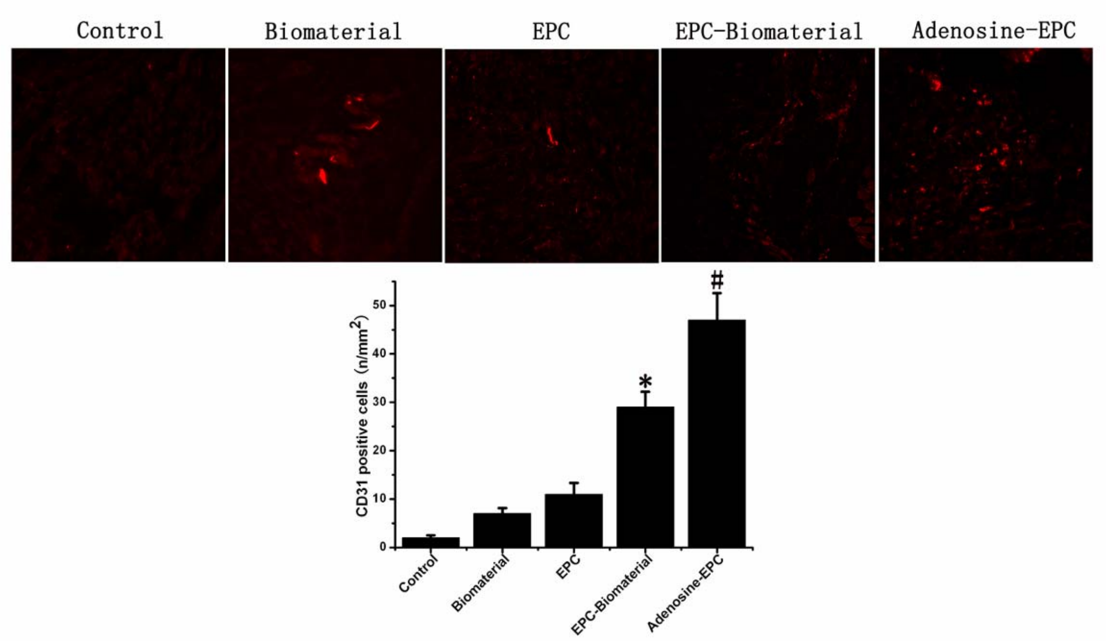

Figure S6. Adenosine inhibited cell apoptosis in diabetic wounds. The results showed number of cell apoptosis in adenosine-EPC group was significantly less than control-EPC group.

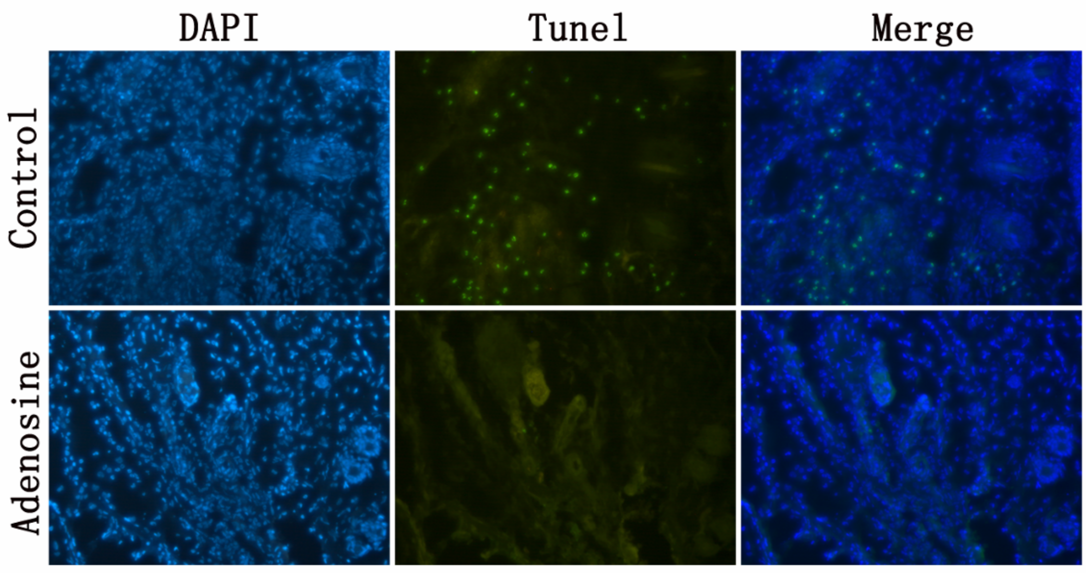

Full-length gels and blots

Figure 1f

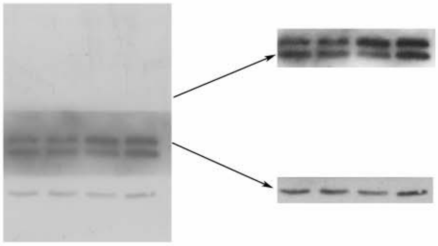

Figure 2a

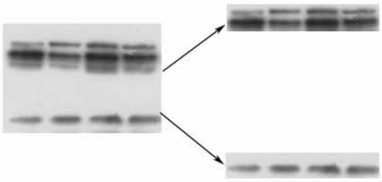

Figure 2a

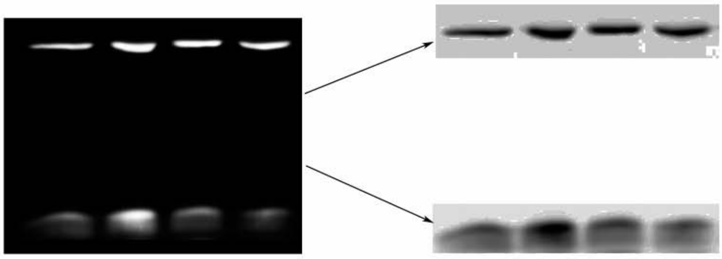

Figure 1f

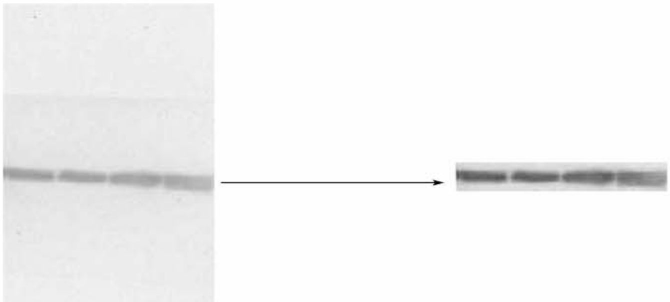

Figure 2a

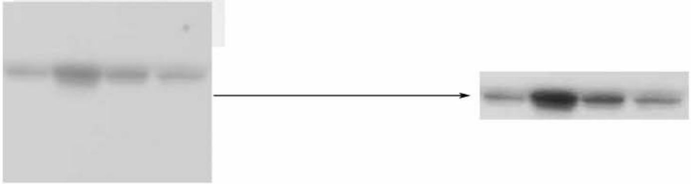

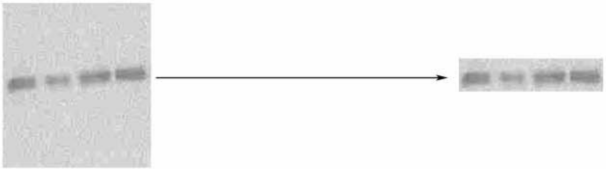

Supplement: Supplementary Information [file srep11594-s1.pdf]
